# Finite-temperature criticality through quantum annealing

**DOI:** 10.1038/s41467-026-75348-3

**Published:** 2026-07-23

**Authors:** Gianluca Teza, Francesco Campaioli, Marco Avesani, Oren Raz

**Affiliations:** 1 Max Planck Institute for the Physics of Complex Systems, Nöthnitzer Str. 38, Dresden, Germany; 2 Department of Physics, University of Trieste, Strada Costiera 11, Trieste, Italy; 3 Department of Physics, School of Science, RMIT University, Melbourne, Victoria Australia; 4 Dipartimento di Fisica e Astronomia G. Galilei, Università degli Studi di Padova, Padova, Italy; 5 Dipartimento di Ingegneria dell’Informazione, Università degli Studi di Padova, Padova, Italy; 6 Padua Quantum Technologies Research Center, Università degli Studi di Padova, via Gradenigo 6B, Padova, Italy; 7 Department of Physics of Complex Systems, Weizmann Institute of Science, Rehovot, Israel

**Keywords:** Quantum simulation, Phase transitions and critical phenomena, Statistical physics

## Abstract

Critical phenomena at finite temperature underpin a broad range of physical systems, yet their study remains challenging due to computational bottlenecks near phase transitions. Quantum annealers have attracted significant interest as a potential tool for accessing finite temperature criticality beyond classical reach, but their utility in precisely resolving criticality has remained limited by noise, hardware constraints, and thermal fluctuations. Here we overcome these challenges, introducing a sampling protocol that combines real-time temperature inference with fine control of the energy scales throughout experiments. A careful embedding strategy allows us to fully capture the finite-temperature critical behavior of the paradigmatic two-dimensional Ising ferromagnet on toroidal lattices up to 2640 spins. By tuning the energy scale of the system and mitigating device defects, we sample effective Boltzmann distributions extracting both the critical temperature and the associated universal critical exponents. Our approach opens the study of equilibrium and non-equilibrium critical phenomena in a broad class of systems at finite temperature.

## Abstract (web summary)

Resolving finite temperature critical behavior near phase transitions is computationally challenging. Here, the authors demonstrate that a carefully calibrated quantum annealer can accurately extract the critical temperature and universal exponents of the two dimensional Ising model at large system sizes.

## Introduction

Finite-temperature critical phenomena refer to the dramatic and universal changes that occur in a physical system as it approaches a phase transition at a non-zero temperature^1^. These phenomena arise across a wide range of settings, from common fluids to extreme conditions. Examples include the fluid critical point where liquid and gas become indistinguishable, the Curie temperature marking the loss of ferromagnetism^2^, and the *λ*-point below which helium exhibits superfluidity^3^. At criticality, properties like correlation length, susceptibility, and heat capacity diverge or become anomalously large. These divergences follow universal scaling laws, defined by critical exponents that depend only on broad features like dimensionality and symmetry, rather than microscopic details^4^. Understanding these phenomena is essential not only for theory but also for applications, where finite-temperature criticality underpins engineered responses such as magnetoresistance^5^, superconductivity^6^, and conductivity modulation across Mott transitions^7^, key for advanced electronic devices.

Decades of progress in theoretical and computational physics have yielded powerful methods for studying critical phenomena, from the celebrated renormalization group theory^8^ to advanced Monte Carlo algorithms^9^. Yet, these methods face persistent challenges. Critical slowing down limits sampling efficiency near critical points^10^, the sign problem hinders simulations of frustrated and fermionic systems^11^, and tensor network approaches struggle with entanglement growth in higher dimensions^12^. These limitations have fueled the exploration of alternative strategies for studying finite-temperature criticality in complex systems.

Recently, quantum computing and simulation platforms have emerged as promising alternatives, with the expectation that quantum coherence and entanglement could open to regimes where classical methods struggle^13,14^. Digital quantum processors have been used to study quantum phase transitions and non-equilibrium critical dynamics^15^, while analog platforms based on cold atoms or trapped ions have successfully realized spin models exhibiting critical behavior^16^.

A particular platform that is gaining attention are quantum annealers. These are analog quantum devices designed to find low-energy configurations of classical spin systems by adiabatically evolving toward a problem-specific Ising Hamiltonian^17^. Operating at finite temperature, they can approximately sample from Boltzmann distributions^18–20^, a feature that has inspired efforts to repurpose them as simulators for the thermodynamics of complex classical and quantum systems^21^. Recent studies have shown that quantum annealers can capture features of classical and quantum phase transitions^22–25^, highlighting their potential for exploring equilibrium statistical mechanics and finite-temperature criticality.

**Fig. 1:**
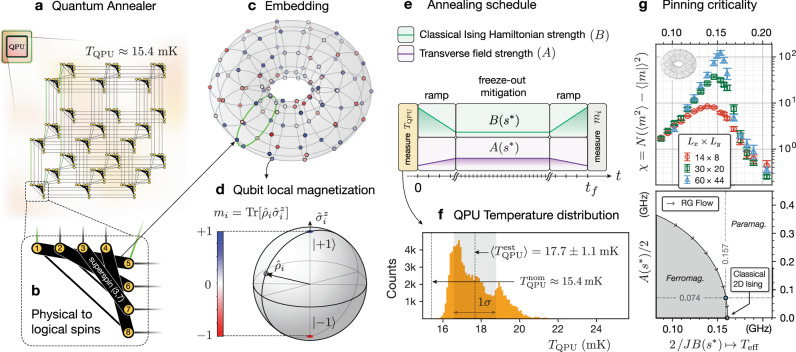
Overview of the embedding and thermal sampling calibration. **a** Quantum processing unit (QPU) of D-Wave’s Advantage system, consisting of a network of qubits cooled to a declared temperature *T*_QPU_ ≈ 15.4 mK, here shown for the embedding of the 2D Ising ferromagnet with toroidal topology. **b** QPU's qubit pairs are strongly coupled to form logical qubits (superspin), enabling periodic boundary conditions. **c** Toroidal 8 × 14 2D Ising ferromagnet associated with the embedding of panel (**a**, **b**). A geodesic on the torus is highlighted in green and shown on the QPU graph. **d** Local qubit state ${\hat{\rho }}_{i}$ of site *i* on the torus, with local magnetization *m*_*i*_, represented on the Bloch sphere. **e** Hardware-encoded annealing schedule: The functions *A*(*s*) and *B*(*s*) determine the transverse and longitudinal energy scales, respectively, as a function of the annealing parameter *s*. Sampling at a fixed intermediate point s^*^ yields equilibrium distributions consistent with a Boltzmann ensemble at some effective temperature *T*_eff_ while preventing the system from getting stuck in an out-of-equilibrium state (freeze-out). **f** Histogram of single-qubit temperature calibration measurements necessary to capture deviations of *T*_eff_. **g** Finite temperature criticality pinned by the magnetic susceptibility *χ*, via the mapping between annealing parameters and the effective temperature *T*_eff_, here represented above a cartoon of the ferromagnet phase diagram. Error bars denote statistical standard errors estimated from the finite number of sampled spin configurations, including spin-gauge realizations.

However, despite their promise, accurately pinning finite-temperature criticality with quantum annealers remains difficult, due to several challenges. Near the critical point, the system’s response time to external changes diverges, demanding impractically long annealing durations^26^. Boltzmann sampling is also affected by fluctuations in the temperature of the quantum processing unit (QPU), illustrated in Fig. 1 a, which is influenced by both external factors and the specific choice of annealing schedules^19^. Furthermore, hardware constraints such as connectivity, coupling noise, and schedule-induced biases affect the reproducibility and reliability of measured observables^27^. Indeed, a recent study has highlighted the challenges of using quantum annealers to probe universal criticality and finite-size scaling when mapping the phase diagram of classical Ising systems of up to 144 spins^25^.

In this work, we tackle these challenges and demonstrate that quantum annealers can be carefully programmed to precisely resolve finite-temperature criticality in complex many-body systems with quantitative control. To benchmark the quality of the approach, we leverage the two-dimensional (2D) ferromagnetic Ising model, thanks to its exactly known critical point^28^. Magnetic leakage, hardware asymmetries, and edge effects are mitigated through a tailored embedding, i.e., mapping between the simulated system and the physical architecture of the annealer, depicted in Fig. 1b, c. A range of effective temperatures is simulated by varying the energy scale of the model, calibrating the QPU’s temperature before each anneal (as shown in Fig. 1e, f). We implement a new sampling protocol that combines real-time temperature inference with a fine control of the energy scales throughout the experiments that provides us with a reliable and reproducible workflow. Altogether, this enables full use of the QPU network to embed lattices with more than 2500 spins. From local observables and correlations — quantified through the magnetization and susceptibility, respectively (Fig. 1d, g) — we extract the critical temperature and obtain finite-size scaling estimates of universal critical exponents.

The ability to measure and control all the relevant parameters of an exactly solvable many body model is extremely useful. We demonstrate some of the corresponding implications in two separate measurements: in the first, we track the magnetization during a non-equilibrium relaxation of the system after a sudden quench through the phase transition in the presence of small longitudinal and transverse magnetic fields. This demonstrates the acceleration gained by the presence of a transverse field. In a second demonstration, we initiate the system in the metastable state associated with all spins pointing against the longitudinal magnetic field. We then measure the probability that the system tunnels through the barrier between the metastable and stable states. Both the barrier height and the transverse field can be controlled independently, shedding light on a key property of quantum annealers, i.e., their ability to tunnel through energy barriers to reach lower-energy states.

Our approach establishes a systematic and scalable method for studying finite-temperature criticality using quantum annealers, applicable to a broad class of classical and quantum systems with embedded Ising representations. By leveraging the tunability of couplings and the relatively high connectivity available on current annealing architectures—now beyond 15 couplings per spin—our methodology extends to frustrated magnets^29^, spin glasses^30^, and constrained models relevant to optimization^31^, and lattice gauge theories^32^. Moreover, the same programmable framework can be adapted to investigate non-equilibrium dynamics and quenched disorder, offering a path for the exploration of critical phenomena in regimes that are challenging for classical methods.

## Results

### Embedding the 2D ising model

In the 2D Ising model, classical spins on a square lattice are coupled with a nearest-neighbors interactions. The model has a disordered phase above its critical temperature. In this phase, the probability to find a spin in each of its state is equal. Below the critical temperature the system has a spontaneously broken symmetry, and most spins point in the same direction. The 2D Ising model is a natural candidate for our purpose due to its well-understood equilibrium properties and its prototypical finite-temperature phase transition. To use this model as a benchmark for a quantum annealer, we begin by embedding the classical 2D Ising ferromagnet onto the hardware graph of the D-Wave Advantage system, which is based on the Pegasus topology^33^.

Directly realizing a regular square lattice with periodic boundary conditions (PBCs) on the Pegasus graph requires careful mapping, as the native qubit connectivity is both sparse and non-planar. We employ a tiling-based embedding strategy that groups strongly coupled physical qubits into logical superspins, as shown in Fig. 1a, b. This approach—used in several previous studies to emulate lattice connectivity and enhance coherence—enables each superspin to behave as a single quantum spin variable, with its internal ferromagnetic couplings set to be significantly stronger than the interactions between superspins^34,35^. It also allows us to impose effective toroidal boundary conditions, shown in Fig. 1c, and mitigate edge effects that would otherwise distort the scaling analysis in finite systems^36^.

We implement ferromagnetic couplings of high amplitude (15–20 times stronger than the interaction between different superspins) to enforce coherence within each superspin. However, such strong couplings can induce magnetic leakage, leading to systematic biases in effective fields experienced by neighboring spins. To correct for these biases, we follow a calibration protocol that introduces local field offsets determined via single-qubit benchmarking procedures^37^. This compensation ensures consistency across different experimental runs and mitigates embedding-related noise. Full details of the shimming procedure and the mitigation of embedding-induced biases are given in the Methods.

Using this method, we successfully embed rectangular lattices with system sizes up to 2640 spins (e.g., 60 × 44), while maintaining the aspect ratio *L*_*x*_/*L*_*y*_ ≈ 3/2 for consistency in scaling analysis. Our largest systems span nearly the full available qubit count on the Advantage system. We constructed multiple such embeddings with randomized gauge transformations (spin-flip symmetries^38^) in each experimental run. Since the ferromagnetic Ising model is invariant under such transformations (upon appropriate decoding), this procedure preserves physical observables while averaging over residual hardware biases^39^. In addition, for the smaller and intermediate system sizes, we implemented multiple embeddings distributed across different regions of the QPU, allowing us to verify robustness against spatial inhomogeneities of the hardware. Further details are provided in the Supplementary Information, and explicit embedding maps are shown in Supplementary Fig. 5.

This embedding strategy forms the foundation for reliable thermal sampling, enabling us to later interpret critical behavior through finite-size scaling and cumulant analysis of sampled spin configurations.

### Finite-temperature sampling

To study equilibrium properties at finite temperature using a quantum annealer, we exploit the device’s ability to sample from thermally populated states near the end of its annealing schedule. The D-Wave system implements a transverse-field Ising model with a time-dependent Hamiltonian:

1 $$\hat{H}(s)=-\frac{A(s)}{2}{\sum}_{i}{\hat{\sigma }}_{i}^{x}+\frac{B(s)}{2}\left[{\sum}_{i}{h}_{i}{\hat{\sigma }}_{i}^{z}+{\sum}_{i > j}{J}_{ij}{\hat{\sigma }}_{i}^{z}{\hat{\sigma }}_{j}^{z}\right],$$

where ${\hat{\sigma }}_{i}^{x}$ and ${\hat{\sigma }}_{i}^{z}$ are Pauli matrices acting on qubit *i*, *h*_*i*_ and *J*_*ij*_ are programmable local fields and couplings, and *s* ∈ [0, 1] parametrizes the annealing schedule. The functions *A*(*s*) and *B*(*s*) control the transverse and longitudinal energy scales and are fixed by the hardware.

At the end of the anneal, the transverse field ideally vanishes and the system is governed by the classical Hamiltonian:

2 $$H(\{{\sigma }_{i}\})=\frac{B(1)}{2}\left[{\sum}_{i}{h}_{i}{\sigma }_{i}+{\sum}_{i > j}{J}_{ij}{\sigma }_{i}{\sigma }_{j}\right],$$

where *σ*_*i*_ = ± 1 and *B*(1) is the final value of the longitudinal energy scale with anneal parameter *s* = 1. If the system remains in contact with thermal environment—the fridge, QPU (quantum processing unit) itself, and other sources—for long enough time, then the sampled spin configurations at the end follow a Boltzmann distribution^40^

3 $$P(\{{\sigma }_{i}\})\propto \exp \left(-\frac{H(\{{\sigma }_{i}\})}{{k}_{B}{T}_{q}}\right),$$

where *T*_*q*_ is the physical temperature of the QPU.

In practice, thermal sampling near *s* = 1 is complicated by the so-called freeze-out phenomenon^41,42^, where the dynamics slow down dramatically and the system becomes trapped in metastable configurations. While the precise onset of freeze-out is not directly encoded in hardware parameters, the annealing schedule shown in Fig. 1e—which defines the evolution of the transverse and longitudinal energy scales via *A*(*s*) and *B*(*s*)—constrains the thermal accessibility of classical configurations. To mitigate freeze-out, we halt the anneal at a control parameter value s^*^ = 0.5, such that *A*(s^*^)/*B*(s^*^) ≈ 0.05 ^33^, ensuring the system remains in a dynamic regime while still allowing for effectively classical readout. Sampling at this intermediate point yields distributions consistent with thermal equilibrium^43^. The non-equilibrium relaxation measurements discussed below provide an auxiliary consistency check that, in the operating regime relevant to this protocol, the system reaches a steady response on experimentally accessible timescales. The primary validation of the choice of s^*^ and equilibrium interpretation used in the criticality analysis is instead provided by the Binder-cumulant crossing and the consistency of the finite-size scaling analysis across system sizes.

A second important issue is temperature calibration^19,44^. The nominal operating temperature of the QPU (${T}_{{{\rm{QPU}}}}^{{{\rm{nom}}}}\approx 15.4$ mK) can fluctuate significantly between experimental runs. These fluctuations are caused by both environmental noise and internal operational procedures, including device programming, readout cycles, and idle state drift. As shown in Fig. 1f, these variations are directly measurable: The histogram displays the distribution of effective QPU temperatures inferred from repeated calibration steps. To compensate for these fluctuations, we perform an in situ temperature estimation before each batch of samples. Specifically, we program single-qubit thermalization problems and extract a maximum-likelihood estimate of their freeze-out temperatures ${T}_{{{\rm{FO}}}}^{{{\rm{est}}}}$ based on excitation probabilities^44^. The value of the anneal control parameter at which the dynamics of this problem freezes out is known (*s*_FO_ = 0.612), and allows us to assess a declared effective temperature at freeze-out of ${T}_{{{\rm{FO}}}}^{{{\rm{nom}}}}=2{k}_{b}{T}_{{{\rm{QPU}}}}^{{{\rm{nom}}}}/B({s}_{{{\rm{FO}}}})\approx 0.164$ (*k*_*B*_ = 1 units). This single-qubit freeze-out measurement is performed across the entire QPU (more than 5 × 10^3^ qubits) with 10^3^ samples, yielding over 5 × 10^6^ data points for each calibration run. Bootstrapping yields a relative uncertainty of order  ~ 10^−3^. Propagating such a variation through the finite-size scaling analysis produces shifts in *T*_*c*_ and in the critical exponents well below the statistical error bars, and can therefore be neglected in the finite-size scaling analysis. The inferred correction factor $\delta={T}_{{{\rm{FO}}}}^{\,{\mbox{est}}\,}/{T}_{{{\rm{FO}}}}^{\,{\mbox{nom}}\,}$ is then used to rescale the classical energy scale *J* across experiments, preserving consistency in the effective sampling temperature. Altogether, this allows us to estimate an average effective temperature of the QPU across all experiments as $< {T}_{{{\rm{QPU}}}}^{{{\rm{est}}}} > =17.7\pm 1.1\,{{\rm{mK}}}$, where the associated error is the variability of the temperature across the experiments.

To our knowledge, this form of real-time temperature compensation has not been applied in previous quantum annealing studies of Boltzmann sampling. Combined with early-termination protocols, it enables consistent finite-temperature sampling across multiple runs, device conditions, and—most importantly—system sizes. Indeed, these steps are fundamental to fully exploit the 5000+ qubits of the QPU^33^, allowing us to embed sizes considerably larger than those used so far for finite-temperature analyses^24,25,45^.

### Pinning criticality via Binder cumulants

To determine whether our annealing protocol successfully prepares the system at the critical point of the 2D Ising model, we analyze the Binder cumulant *b*_*N*_(*J*) as a function of the ferromagnetic coupling strength *J*^46^. The Binder cumulant is defined via moments of the total magnetization *M* = ∑_*i*_*σ*_*i*_:

4 $${b}_{N}(J)=\frac{1}{2}\left(3-\frac{\langle {M}^{4}\rangle }{{\langle {M}^{2}\rangle }^{2}}\right),$$

where the averages are taken over samples generated for a system of *N* spins at fixed *J*. This quantity is sensitive to the shape of the magnetization distribution: it approaches 0 in the disordered phase (Gaussian fluctuations), 1 in the ordered phase (double-peak distribution), and takes an intermediate value. At the critical point, where the magnetization scaling function is non-Gaussian^47–49^, the Binder cumulant approaches a size-independent value for fixed boundary conditions, aspect ratio, and topology^50^.

**Fig. 2:**
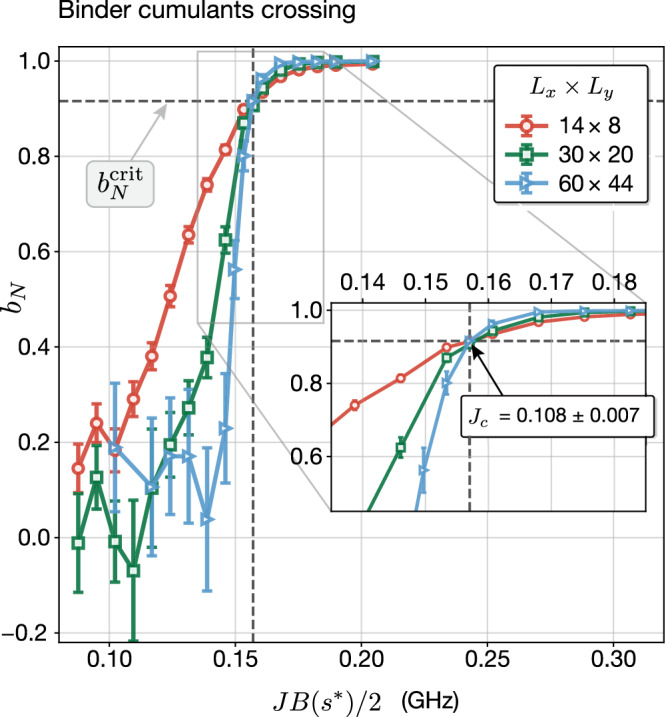
Pinning finite-temperature criticality. Binder cumulant *b*_*N*_(*J*) for three system sizes (*N* = 112, 600, 2640) as a function of the effective ferromagnetic coupling *J*. The curves cross at *J*_*c*_ = 0.108 ± 0.007, indicating the presence of a critical point in the thermodynamic limit (inset). The crossing value *b*_*N*_(*J*_*c*_) = 0.91 ± 0.04 matches the known value for a 2D Ising model with periodic boundary conditions and aspect ratio *L*_*x*_/*L*_*y*_ ≈ 3/2. This validates the effectiveness of the sampling protocol in recovering critical behavior. Error bars denote statistical standard errors estimated from sampled spin configurations, including spin-gauge realizations; uncertainties on *J*_*c*_ and *b*_*N*_(*J*_*c*_) are standard deviations obtained with the bootstrap procedure detailed in the Methods.

We perform annealing runs for three system sizes—*N* = 112, 600, and 2640 spins—chosen to preserve an approximate aspect ratio *L*_*x*_/*L*_*y*_ ≈ 1.5 (Specifically, we used (*L*_*x*_, *L*_*y*_) ∈ {(14, 8), (30, 20), (60, 44)} corresponding to 1.75, 1.5 and  ≈ 1.36). Each run samples spin configurations for a range of effective couplings *J*, as adjusted through our temperature-calibrated protocol. The resulting Binder cumulants are shown in Fig. 2. Remarkably, the cumulant curves for all system sizes cross at a common coupling value *J*_*c*_ = 0.108 ± 0.007, strongly indicating that our sampling ensemble lies at the thermodynamic critical point. The critical point at unit temperature is exactly known^28^, and an estimate of the critical temperature in the presence of a transverse field can be obtained through quantum Monte Carlo simulations^51^. Our sampling schedule provides direct control over the energy scales at play when measurements are performed, enabling us to provide direct estimates of physical and effective temperatures. Therefore, we can provide here an estimate of the critical temperature for the 2D Ising model of ${T}_{{{\rm{crit}}}}=2{k}_{B}{T}_{{{\rm{QPU}}}}^{{{\rm{nom}}}}/({J}_{{{c}}}B({s}^{*}))=2.04\pm 0.13$ in the presence of a transverse field $A({s}^{*})/(2{k}_{B}{T}_{{{\rm{QPU}}}}^{{{\rm{nom}}}})\approx 0.23$ (*k*_*B*_ = 1 units), in line with quantum Monte Carlo predictions (see^51^). While deviations from the expected value could be ascribed to the use of superspins in the embedding (see Fig. 1b), it is important to observe that the observed crossing value *b*_*N*_(*J*_*c*_) = 0.91 ± 0.04 closely matches Monte Carlo benchmarks for the 2D Ising model with periodic boundary conditions and *L*_*x*_/*L*_*y*_ = 1.5 ^52^, confirming that our annealing procedure produces correct critical fluctuations despite the hardware limitations that constrained the Binder-cumulant analysis in Ref. ^25^. To robustly estimate the uncertainties on *J*_*c*_ and *b*_*N*_(*J*_c_), we perform a bootstrap resampling procedure consistent with the corresponding experimental uncertainties (see “Methods” and Supplementary Information)^53^.

This cumulant analysis provides a key validation of our sampling and embedding procedures, demonstrating that they yield thermal ensembles at effective temperature scales compatible with known classical criticality. The accuracy of the cumulant crossing further supports our choice of s^*^ and the robustness of our in situ temperature correction strategy.

### Critical exponents and finite-size scaling

To further characterize the universality class of the observed transition and quantify the consistency of our sampling protocol, we extract critical exponents from finite-size scaling of magnetization and susceptibility. We focus on the standard order parameter exponent *β*, susceptibility exponent *γ*, and correlation length exponent *ν*^54^.

We define the reduced coupling constant relative to the critical point as *τ* = (*J*_*c*_ − *J*)/*J* and express scaling hypotheses for observable quantities. The absolute magnetization density 〈∣*m*∣〉 = 〈∣*M*∣〉/*N* is expected to scale as

5 $$\langle | m| \rangle={L}^{-\beta /\nu }{f}_{m}(\tau {L}^{1/\nu }),$$

and the susceptibility *χ* = *N*(〈*m*^2^〉 − 〈∣*m*∣〉^2^) should obey

6 $$\chi={L}^{\gamma /\nu }{f}_{\chi }(\tau {L}^{1/\nu }),$$

where *f*_*m*_ and *f*_*χ*_ are universal scaling functions, and $L=\sqrt{N}$ is the effective linear system size. These expressions follow from the finite-size scaling hypothesis, which posits that near criticality, observable quantities depend only on the combination *τL*^1/*ν*^^55^. This framework underlies the expected collapse behavior for magnetization and susceptibility in finite systems^56^.

The critical exponents of the 2D Ising model are well-established, with exact values given by $\beta=\frac{1}{8}$, $\gamma=\frac{7}{4}$, and *ν* = 1 ^28^. These values serve as a benchmark for validating the consistency of our sampling protocol and the fidelity of critical fluctuations.

**Fig. 3:**
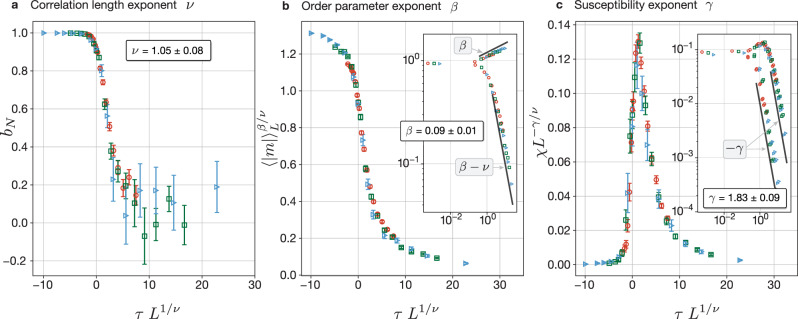
Recovering the critical exponents. **a** Plotting the Binder cumulant against the reduced temperature $\tau =(J_{c}-J)/J$ rescaled by *L*^1/*ν*^, where *ν* is the correlation-length exponent and $L=\sqrt{{L}_{x}\times {L}_{y}}$. The plot shows an excellent collapse around the critical region τ=0 for the extracted value *ν* = 1.05 ± 0.08, in agreement with the expected value for a 2D Ising ferromagnet at criticality. **b** Collapse of the rescaled order parameter $< | m| > {L}^{\beta /\nu }$ shows an excellent agreement of the data for the three different system sizes *N*, yielding an extracted order parameter exponent *β* = 0.09 ± 0.01. Visualization in log-scale shows perfect agreement with the power-law behavior  ~ *β* − *ν* ( ~ *β*) for the disordered (ordered) branch. **c** Collapse of the rescaled susceptibility *χL*^−*γ*/*ν*^ shows a strong agreement with the different system sizes and a good agreement of the extracted exponent *γ* = 1.83 ± 0.09 with the expected value. Visualization in log-scale shows perfect agreement with the power-law behavior  ~ − *γ* for both τ ≶ 0 branches. In all panels, error bars denote statistical standard errors estimated from sampled spin configurations, including spin-gauge realizations; quoted exponent uncertainties are standard deviations obtained with the bootstrap procedure detailed in the Methods.

Figure 3a illustrates the scaling collapse for the three system sizes used in our Binder cumulant analysis. Panel **b** shows the magnetization collapse while panel **c** displays the susceptibility collapse^57^. In both cases, we observe excellent collapse over a broad range of *τL*^1/*ν*^, especially near the critical region. The consistency and sharpness of the collapse across more than a decade in system size provide strong evidence that our sampling ensembles reflect equilibrium criticality. To quantify this agreement and extract precise values of the critical exponents, we employed a technique to optimize the finite-size collapse^58^ (see “Methods”). Through a bootstrap analysis, we obtain the following exponents from the data: *ν* = 1.05 ± 0.08, *β* = 0.09 ± 0.01, and *γ* = 1.83 ± 0.09. These values show strong consistency with the theoretical values for the 2D Ising model^28^, with *ν* and *γ* in excellent agreement within 1*σ* of their theoretical values, and *β* deviating only slightly from the expected value. This agreement supports the identification of the observed transition with the Ising universality class. Importantly, the high quality of the data collapse represents a substantial improvement over earlier studies, which were often limited by smaller lattices, open boundary conditions, or uncontrolled thermal drift. To better understand the deviation of *β* from its theoretical value, we compare the experiment to a Monte Carlo simulation of the classical 2D Ising model on the same lattice sizes (see Methods, Supplementary Fig. 2, and Supplementary Fig. 3). As in the experimental results, the values of *γ* and *ν* are consistent with the theoretical values, whereas the measured value of *β* is about 2*σ* lower. This suggests that the variations in the lattice aspect ratio and limited statistics, together with the relatively small value of the exponent, are the main reasons for the deviations in *β*.

We emphasize that this scaling analysis is made possible by the consistency of the sampling protocol across both system sizes and annealing sessions together with the real-time temperature calibration routine, which allows us to remove systematic biases. The high quality of data collapse reflects not only the statistical fidelity of the samples but also the effectiveness of our protocol in stabilizing temperature and avoiding freeze-out effects. Indeed, employing a standard anneal protocol with a linear ramp in the control parameter *s* exposes the experiments to considerable uncertainty in the precise value of the parameters at sampling times as well as different behaviors across system sizes – hindering finite-size scaling analysis and extraction of critical exponents. Therefore, compared to standard sampling procedures employed so far in the literature, the real-time temperature inference combined with a controlled quench at constant energy parameters is what enables reliable control of experiments across system sizes, make use of the full QPU network and ultimately reconnect to physically meaningful temperatures through the controlled energy scales.

### Quantum annealing speedup near criticality

Quantum annealing (QA) is often benchmarked by its ability to reach true ground states in complex energy landscapes^59,60^, where metastable states separated by large barriers challenge quantum and classical methods alike. While a full understanding of the transition towards a spin-glass phase is still missing^61,62^, a recent work highlighted how, in the presence of certain symmetries, quantum annealing can still provide an advantage over classical methods when crossing a critical line^63^, both in terms of accuracy and anneal time.

Here, we test QA’s ability to resolve such barriers and escape from non-equilibrium conditions by initializing the system in either disordered or metastable configurations. In both cases, we consider a transverse-field Ising Hamiltonian with a longitudinal bias field,

7 $$\hat{H}=-J\left({\sum}_{\langle i,j\rangle }{\sigma }_{i}^{z}{\sigma }_{j}^{z}+\epsilon {\sum}_{i}{\sigma }_{i}^{z}\right)-\Gamma {\sum}_{i}{\sigma }_{i}^{x},$$

where *J* is the Ising interaction strength between nearest-neighbor spin pairs 〈*i*, *j*〉, *Γ* is the transverse field, and *Jϵ* is a uniform longitudinal field that biases the system toward a symmetry-broken state.

**Fig. 4:**
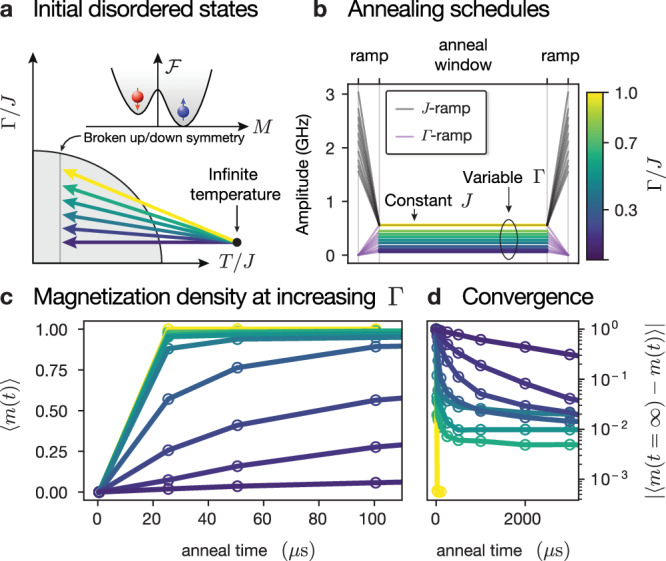
Annealing speedup near criticality. **a** A schematic illustration of the experiment of panel b: a random configuration (corresponding to infinite temperature—paramagnetic phase) is quenched to Ising 2D Hamiltonian with various strength of the transverse field small enough to keep the system in the ferromagnetic phase. **b** Anneal schedules to emulate evolutions at constant effective temperatures and different transverse fields amplitudes. Here *J*/*T* ≈ 0.9, *Γ*/*J* ∈ [0.1, 1] and a small longitudinal field (*ϵ* = 0.01) breaks the symmetry favoring the positive direction. **c** Magnetization density evolution <m(t)> for several transverse field amplitudes *Γ* (system size *N* = 2640). Larger *Γ* values lead to a faster convergence, fundamental to ensure steady-state convergence within the maximum annealing window. **d** Convergence of the magnetization towards the steady state value <m(t=∞)>. Failing to converge for small enough transverse field amplitudes is related to single realizations thermalizing in the wrong (metastable) well. Therefore, larger *Γ* values increase reliability in the convergence to the symmetry-broken ground state.

First, we investigate how a disordered spin configuration evolves under a fixed Hamiltonian with nonzero transverse and longitudinal fields. This setup mimics a quench from an infinite-temperature state to a low-temperature effective Hamiltonian, as shown in Fig. 4a, allowing us to probe the system’s relaxation dynamics and the nature of its final state. To do so, we initialize the system in a random spin configuration and quench to a transverse-field Ising Hamiltonian with fixed *Γ* and *ϵ* = 0.01. The system was allowed to evolve for varying durations, and the average longitudinal magnetization was recorded as a function of time. This experiment was repeated for several values of the transverse field *Γ*, as shown in Fig. 4b.

The results, shown in Fig. 4c, d, indicate that at small transverse fields, the relaxation is slow and the magnetization saturates at a value significantly below the equilibrium expectation. This indicates that the system often becomes trapped in metastable states, failing to reach the true symmetry-broken minimum on the timescale of the experiment. As the transverse field increases, relaxation becomes faster and the saturation magnetization approaches the expected ground-state value.

**Fig. 5:**
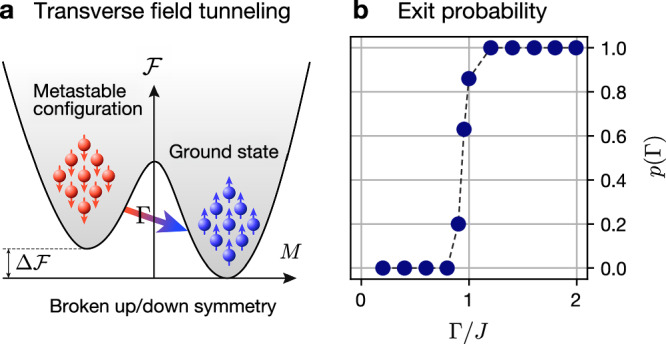
Barrier crossing via transverse field. **a** A schematic illustration of the experiment of panel b: the system is prepared in the metastable configuration (corresponding to all-down spins) of the Ising 2D Hamiltonian (Eq. (7)), with *T*/*J* ≈ 1.1, *ϵ* = 0.05 (system size *N* = 2640). Increasing the transverse field amplitude—while staying in the ferromagnetic phase (*Γ*/*J* ∈ [0.2, 2])—should eventually enable the crossing of the barrier and lead the system to the correct ground state. **b** Probability *p*(*Γ*) of successful reversal from the metastable (all-down) configuration to the symmetry-breaking ground state as a function of *Γ*. The sharp change indicates an enhanced ability to escape metastable states due to the transverse field.

We then consider a setting in which the system is initialized in a fully polarized state opposite to the direction favored by the longitudinal field, i.e., a metastable configuration, as illustrated in Fig. 5a. The system is quenched to the transverse-field Ising Hamiltonian of Eq. (7), with a fixed longitudinal bias of *ϵ* = 0.05 and varying values of the transverse field *Γ*. After a hold time of 1000 *μ*s, the spin configuration is measured, and the probability of reaching the correct symmetry-broken ground state is recorded as a function of *Γ*.

The results, shown in Fig. 5b, indicate that for *Γ*/*J* ≪ 1 the system remains trapped in the metastable state within the anneal timescale. In this regime, the microscopic quantum dynamics of magnetization domains is perturbatively coherent^64,65^, supporting the stability we observe. As *Γ* increases, the success probability rises sharply around *Γ*/*J* = 1, signaling the onset of barrier crossing. This transition reflects the role of the transverse field in facilitating escape from metastability. Plugging the experimental parameters (*J* = 0.3212 GHz, *ϵ* = 0.05) with the observation that the transition happens at *Γ/J* ≈ 1, implies that the system is able to tunnel through a barrier height of ~80 *μ*eV, which is roughly 60 times larger than the thermal energy of 17 mK. To distinguish between quantum tunneling and classical hopping over the barrier due to thermal fluctuations, in the Supplementary Information we report additional experiments where both *J* and *ϵ* were varied in combinations that keep the classical barrier height fixed. The corresponding relaxation fractions are reported in Supplementary Table 1, showing that the transverse field is crucial for the relaxation at a constant barrier height. This provides strong evidence against a purely classical thermal interpretation and is consistent with a substantial quantum contribution to the relaxation mechanism.

Let us note that the values of *Γ* explored in the experiment remain well below the critical transverse field *Γ*_*c*_ at which the ordered phase disappears and the free energy barrier vanishes altogether^66^. The zero-temperature quantum critical point for the transverse-field Ising model on the square lattice occurs at *Γ*_*c*_/*J* ≈ 3.044, and in our experiment, where *T*/*J* ≈ *1.1* the critical transverse field is bounded by *Γ*_*c*_/*J* > 2.5^51^. Here, the maximum value of *Γ* used corresponds to *Γ*/*J* ≈ 2, and the effective temperature remains well below the thermal phase boundary. Therefore, we are operating in a regime where symmetry-broken states remain metastable minima of the free energy landscape, and the concept of a barrier between them is still well-defined. This ensures that the observed transition from trapping to escape reflects a meaningful crossing (or tunneling) event between locally stable configurations, rather than a smooth crossover between disordered states.

## Discussion

In this work, we demonstrated that quantum annealers, when carefully calibrated, can accurately resolve finite-temperature criticality in complex, large-scale many-body systems. The calibration of QPU temperature and mitigation of freeze-out effects proved essential for reliably extracting the critical temperature and critical exponents of the 2D Ising ferromagnet up to 2640 spins. While hardware limitations (native topology of the QPU, freeze-out point) have been regarded so far as bottlenecks—even in more recent architectures^25,33^—our operational line bypasses these limitations, enabling reliable analysis for finite-temperature criticality in both current and older architectures. This innovative sampling protocol combines real-time temperature inference with fine control of the energy scales, paving a practical way to the usage of quantum annealers as quantum simulators to study a broad range of phenomena in and outside equilibrium. Thanks to our embedding strategy, our approach extends to a broad class of systems and problems that map to the Ising model. This includes quantum gravity models^67,68^, non-equilibrium quantum thermodynamics, e.g., charging and work extraction in quantum Ising networks^69,70^, anomalous relaxation phenomena^71–74^, e.g., quantum Mpemba effect^75–77^, and quantum circuit compilation^78^. Further work on this approach should address the issue of memory effects (non-Markovianity) that occur between quantum annealing schedules^79^. Finally, systematic comparisons with classical algorithms could further clarify the nature and extent of any potential advantage of quantum sampling or optimization near criticality.

## Methods

### Shimming and mitigation of embedding-induced biases

The implementation of a 2D lattice on the quantum annealer relies on superspin embeddings, in which each logical spin is realized by a chain of strongly coupled physical qubits. While this construction provides the desired connectivity, it also amplifies the impact of device imperfections: stray local fields and residual coupler crosstalk induce effective longitudinal fields on the physical qubits, producing magnetic leakage at the level of the superspins. If uncompensated, these effects introduce correlated biases that distort the measured observables. To mitigate them, we employ a shimming procedure that calibrates the flux-bias offsets (FBOs) of the physical qubits, following the general strategy of Ref. ^37^, but performed at the level of the superspins rather than at the level of individual physical qubits. In practice, the feedback is built from the magnetization of the logical degrees of freedom, allowing the procedure to compensate the effective fields generated by the embedding itself, including magnetic leakage. The shimming is implemented through an iterative feedback scheme. At each iteration, we estimate the local magnetization ${m}_{i}=\langle {\sigma }_{i}^{z}\rangle$ of each superspin and compare it to the bulk average $\bar{m}$, computed over bulk sites to avoid boundary effects. The flux-bias offsets are then updated according to the gradient-descent rule

8 $${{{\Phi }}}_{i}^{(n+1)}={{{\Phi }}}_{i}^{(n)}-\alpha \left({m}_{i}-\bar{m}\right),$$

where *α* is a small learning rate. The calibration is performed with the full problem Hamiltonian in place, so that it compensates the effective fields in the actual operating conditions of the experiment. We further suppress systematic biases by randomizing the implemented problem through gauge transformations (problem isomorphisms). At each experimental run, random variables *g*_*i*_ = ± 1 are assigned, transforming couplings and fields as *J*_*ij*_ → *g*_*i*_ *g*_*j*_ *J*_*ij*_ and *h*_*i*_ → *g*_*i*_*h*_*i*_, and configurations are decoded through *σ*_*i*_ → *g*_*i*_*σ*_*i*_. Since the ferromagnetic Ising model is invariant under this transformation, the procedure preserves physical observables while averaging out residual device-dependent biases^38,39^. The convergence of the FBOs and the explicit embeddings used for each lattice size are reported in Supplementary Fig. 4 and Supplementary Fig. 5. We have verified that, after these procedures, the observables entering the finite-size scaling analysis are stable across different embeddings, QPU regions, and gauge realizations, so that residual imperfections do not bias the extracted critical behavior.

### Estimation of experimental parameters and uncertainties

The critical point is determined from the fourth-order Binder cumulant *b*_*N*_(*J*) measured for the three system sizes *N* = 112, 600, and 2640. For each size we generate a continuous representation of *b*_*N*_(*J*) by interpolation, and locate the crossing as the coupling that minimizes the dispersion between the three curves, i.e. that minimizes

9 $$f(J)={\sum }_{i=1}^{3}{\left({b}_{{N}_{i}}( \, J)-\bar{b}( \, J)\right)}^{2},\quad \bar{b}(J)=\frac{1}{3}{\sum }_{i=1}^{3}{b}_{{N}_{i}}( \, J).$$

The minimizing coupling is our estimate of *J*_*c*_, and the critical Binder cumulant is ${b}_{N}({J}_{{{c}}})=\bar{b}({J}_{c})$. Uncertainties on *J*_*c*_ and *b*_*N*_(*J*_*c*_) are obtained by a non-parametric bootstrap^53^: we generate 10^4^ synthetic datasets, drawing each data point from a normal distribution centered on its measured value with standard deviation equal to its experimental uncertainty, repeat the full analysis on each, and take the standard deviation of the resulting distribution as the uncertainty. The universal exponents *ν*, *β*, and *γ* were extracted by finite-size scaling using a quantitative measure of data collapse^58^. For a trial exponent *θ*, the interpolated scaled data for size *L*_*i*_ define a function *g*_*i*_(*x*; *θ*), and the quality of the collapse between two sizes is measured over their common range by

10 $${\sigma }_{ij}^{2}(\theta )=\frac{1}{{x}_{\max }-{x}_{\min }}{\sum }_{{x}_{\min }}^{{x}_{\max }}{[{g}_{i}(x;\theta )-{g}_{j}(x;\theta )]}^{2},$$

with total cost $S(\theta )={\sum }_{i < j}{\sigma }_{ij}^{2}(\theta )$ minimized over *θ*. The exponents are determined sequentially for stability: *ν* is fixed first from the Binder-cumulant collapse (using the optimized *J*_*c*_), and then, holding *ν* fixed, *β* and *γ* are obtained from the magnetization and susceptibility collapses, respectively. Their uncertainties are estimated with the same bootstrap procedure, propagating the uncertainty on *J*_*c*_ (and on *ν* for *β* and *γ*).

### Classical Monte Carlo baseline

To assess whether the small deviations in the extracted exponents arise from finite-size and analysis-window effects, we performed independent classical Monte Carlo simulations of the 2D ferromagnetic Ising model on the same lattice geometries (*L*_*x*_, *L*_*y*_) ∈ {(14, 8), (30, 20), (60, 44)} with periodic boundary conditions, coupling *J* = 1, and zero longitudinal field. Configurations were generated by single-spin Metropolis updates with random-site selection, accepting a proposed flip with probability $\min \{1,{e}^{-\beta \Delta E}\}$. For each size and temperature on a grid refined near *T*_*c*_, we generated *N*_MC_ = 10^4^ independent realizations, each initialized in a uniform configuration and evolved for *N*_sweeps_ sweeps, recording *m*, ∣*m*∣, *m*^2^, and *m*^4^ at the final sweep. To match the experimental readout statistics, which differ across sizes, we downsampled the Monte Carlo pool to the experimental read counts (${N}_{\exp }=1000,\,400,\,100$ for the three sizes) and used *R* = 5000 bootstrap resamples to define the error bars. Applying the same finite-size collapse pipeline as for the experimental data yields *ν* = 0.99 ± 0.06, *β* = 0.110 ± 0.007, and *γ* = 1.66 ± 0.09, with compatibilities to the exact 2D Ising values of 0.16*σ*, 2.11*σ*, and 1.01*σ*, respectively. The corresponding collapses and bootstrap distributions are shown in Supplementary Fig. 2 and Supplementary Fig. 3. The close agreement of both the central values and the uncertainties between the classical baseline and the experiment—in particular the comparable  ~ 2*σ* underestimate of the small exponent *β*—indicates that the experimental data are consistent with the 2D Ising universality class, and that the deviation of *β* originates from finite statistics and the small variation in lattice aspect ratio rather than from hardware effects.

## Supplementary information

Supplemental Information

Transparent Peer Review file

## Data Availability

The data generated in this study have been deposited at (https://github.com/gteza/finite-temperature-criticality-dwave).

## Code availability

The code supporting the findings of this study has been deposited at (https://github.com/gteza/finite-temperature-criticality-dwave), including materials related to the superspin embeddings, shimming procedure, and spin-gauge transformations.
