# Supplementary material for: Finite-temperature criticality through quantum annealing
Source: Nat Commun. 2026 Jul 23;17:7571. doi: 10.1038/s41467-026-75348-3 (PMC13415517; doi:10.1038/s41467-026-75348-3)
Supplement: Supplementary file 1 — Supplemental Information [file 41467_2026_75348_MOESM1_ESM.pdf]

Supplementary Information for: “Finite-temperature criticality through quantum annealing”

Gianluca Teza,[1,2,a] Francesco Campaioli,[3,4,b] Marco Avesani,[5,6,c] and Oren Raz[7,d]
[1] *Max Planck Institute for the Physics of Complex Systems, Nöthnitzer Str. 38, 01187 Dresden, Germany*
[2] *Department of Physics, University of Trieste, Strada Costiera 11, 34151 Trieste, Italy*
[3] *Department of Physics, School of Science, RMIT University, Melbourne, Victoria, Australia*
[4] *Dipartimento di Fisica e Astronomia G. Galilei, Universitá degli Studi di Padova, 35131 Padova, Italy*
[5] *Dipartimento di Ingegneria dell'Informazione, Universitá degli Studi di Padova, 35131 Padova, Italy*
[6] *Padua Quantum Technologies Research Center, Università degli Studi di Padova, via Gradenigo 6B, IT-35131 Padova, Italy*
[7] *Department of physics of complex systems, Weizmann institute of science, Rehovot, Israel, 76100*

(Dated: 24 June 2026)

I. DERIVATION OF THE NUCLEATION BARRIER IN THE 2D TRANSVERSE-FIELD ISING MODEL

This section provides the derivation and physical justification for the expression used to estimate the nucleation barrier between metastable and stable states in the two-dimensional transverse-field Ising model (TFIM) at zero temperature.The formula used in the main text is rooted in a semi-classical extension of classical nucleation theory via a Suzuki–Trotter mapping of the quantum model to an effective (2+1)-dimensional classical model[1].

A. Physical model

We consider the TFIM Hamiltonian with a small longitudinal field:

$$\hat{H} = -J \left(\sum_{\langle ij \rangle} \hat{\sigma}_i^z \hat{\sigma}_j^z + \epsilon \sum_i \hat{\sigma}_i^z \right) - \Gamma \sum_i \hat{\sigma}_i^x,$$

where:

- $J > 0$ is the ferromagnetic coupling,
- Γ is the transverse field introducing quantum fluctuations,
- $\epsilon \ll 1$ is a small longitudinal bias field selecting one magnetization sector.

For the following estimation, the system is taken at zero temperature ($T = 0$) with $\Gamma < \Gamma_c$, where $\Gamma_c \approx 3.04J$ marks the quantum critical point beyond which long-range order disappears[2,3].

B. Suzuki–Trotter mapping

To analyze quantum nucleation in the 2D TFIM, we use the Suzuki–Trotter decomposition to map the quantum model at inverse temperature β to a classical Ising model in (2+1) dimensions. In this mapping, the original two spatial dimensions remain intact, while the imaginary-time dimension is discretized into $M = \beta/\Delta\gamma$ slices, where

[a] Electronic mail: teza@pks.mpg.de
[b] Electronic mail: francesco.campaioli@rmit.edu.au
[c] Electronic mail: marco.avesani@unipd.it
[d] Electronic mail: oren.raz@weizmann.ac.il

$\Delta\gamma$ is the Trotter time step. The resulting classical model is defined on a cubic lattice of size $L \times L \times M$, with anisotropic nearest-neighbor couplings:

$$K = J\Delta\gamma, \quad K_\gamma = -\frac{1}{2}\log\tanh(\Gamma\Delta\gamma),$$

corresponding to the spatial and temporal directions, respectively.

The longitudinal field $J\epsilon$ in the quantum model becomes an effective field $J\epsilon\Delta\gamma$ per time slice in the classical model. In this framework, quantum tunneling processes are interpreted as thermally activated droplet formation in Euclidean spacetime.

C. Calibrating the model

Although the Suzuki–Trotter mapping becomes exact in the limit $\Delta\gamma \to 0$, we adopt a finite value of $\Delta\gamma$ for practical and conceptual convenience. Since we work in the zero-temperature limit $T \to 0$, the number of Trotter slices $M = \beta/\Delta\gamma$ diverges even at fixed $\Delta\gamma$, ensuring convergence of the mapping.

To calibrate $\Delta\gamma$, we use the fact that the mapped (2+1)-dimensional classical Ising model becomes critical when its spatial coupling equals the known critical coupling of the 3D classical Ising model:

$$K = J\Delta\gamma = K_c^{(3\mathrm{D})} \approx 0.2216.$$

Solving for $\Delta\gamma$, we find:

$$\Delta\gamma = \frac{K_c^{(3\mathrm{D})}}{J}.$$

Using the experimental value $J \approx 0.3212\,\mathrm{GHz}$, this gives:

$$\Delta\gamma \approx \frac{0.2216}{0.3212} \approx 0.69\,\mathrm{ns}.$$

This calibrated time step ensures that the spatial coupling in the mapped 3D model reaches the classical critical point when the 2D TFIM reaches its known quantum critical value $\Gamma_c \approx 3.04J$. The temporal coupling at this point is then:

$$K_\gamma(\Gamma_c, \Delta\gamma) = -\frac{1}{2}\log\tanh(\Gamma_c\Delta\gamma),$$

which can be verified to also approach $K_c^{(3\mathrm{D})}$, confirming consistency of the mapping.

D. Semi-classical droplet action

We analyze the nucleation of a droplet of the stable phase (aligned with h) within a metastable background. Near $T = 0$, such nucleation occurs via quantum tunneling, modeled as the formation of a compact droplet in 3D Euclidean space-time.

The action cost $\Delta\mathcal{S}(R, \ell)$ for a cylindrical droplet of spatial radius R and temporal extent ℓ is:

$$\Delta\mathcal{S}(R, \ell) = 2\pi R\ell \cdot \sigma + \pi R^2 \cdot \sigma_\gamma - \pi R^2 \ell \cdot \Delta f,$$

where:

- σ is the spatial domain wall tension, i.e., the energy cost per unit length of domain wall in space. At $\Gamma = 0$, it takes the classical value $\sigma_0 = 2J$ in lattice units. For $\Gamma > 0$, quantum fluctuations soften domain walls, and we model this as $\sigma(\Gamma) = \sigma_0\,(1 - \Gamma/\Gamma_c)^\mu$.

- σ_γ is the temporal wall tension, representing the action cost per unit area of the droplet caps in imaginary time. It arises from the transverse field Γ, and is given by $\sigma_\gamma(\Gamma) = -\frac{1}{2}\log\tanh(\Gamma\Delta\gamma)$. As expected, it vanishes at $\Gamma = 0$, where no tunneling occurs.

- $\Delta f = 2m(\Gamma)J\epsilon$ is the energy density difference due to the longitudinal field,

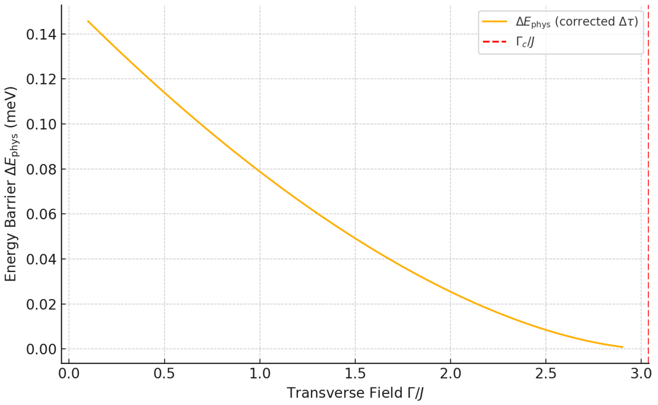

Supplementary Figure 1. Barrier height as a function of Γ, estimated using nucleation theory for $\epsilon = 0.05$ and $J \approx 0.3212\,\mathrm{GHz}$.

- $m(\Gamma)$ is the spontaneous magnetization at transverse field Γ.

Extremizing this action with respect to R and ℓ gives the critical droplet configuration that dominates the tunneling process. The partial derivatives are:

$$\frac{\partial \Delta \mathcal{S}}{\partial \ell} = 2\pi R \cdot \sigma - \pi R^2 \cdot \Delta f = 0 \quad \Rightarrow \quad R_c = \frac{2\sigma}{\Delta f},$$

$$\frac{\partial \Delta \mathcal{S}}{\partial R} = 2\pi \ell \cdot \sigma + 2\pi R \cdot \sigma_\gamma - 2\pi R \ell \cdot \Delta f = 0 \quad \Rightarrow \quad \ell_c = \frac{\sigma_\gamma}{\Delta f}.$$

The physical energy barrier is then given by the dimensionful expression:

$$\Delta E_{\rm phys}(\Gamma, \epsilon) = \frac{\hbar \Delta \mathcal{S}_c}{\ell_c \Delta \gamma} = \frac{2\pi \hbar \sigma^2(\Gamma)}{\Delta\gamma \cdot m(\Gamma) J \epsilon}.$$

We model the dependencies of σ and m on the transverse field using scaling forms:

$$\sigma(\Gamma) = \sigma_0 \left(1 - \frac{\Gamma}{\Gamma_c}\right)^{\mu}, \quad m(\Gamma) = \left(1 - \frac{\Gamma}{\Gamma_c}\right)^{\beta},$$

where $\sigma_0 = 2J$, $\mu \approx 1$ (see[4]), and $\beta \approx 0.32$ (see[5]). Substituting the empirical scaling forms and using $\sigma_0 = 2J$, we obtain:

$$\Delta E_{\rm phys}(\Gamma, \epsilon) = \frac{2\pi\hbar (2J)^2}{\Delta\gamma \cdot J\epsilon} \cdot \left(1 - \frac{\Gamma}{\Gamma_c}\right)^{2\mu - \beta} = \frac{8\pi\hbar J}{\Delta\gamma \cdot \epsilon} \cdot \left(1 - \frac{\Gamma}{\Gamma_c}\right)^{2\mu - \beta} = \frac{8\pi\hbar J^2}{K_c^{(3\mathrm{D})} \epsilon} \cdot \left(1 - \frac{\Gamma}{\Gamma_c}\right)^{2\mu - \beta}.$$

All quantities in this expression are in physical units, and $\Delta E_{\rm phys}$ has units of energy.

In the relevant experimental conditions in the main text, we used $\epsilon = 0.05$ and $J \approx 0.3212\,\mathrm{GHz}$. For these values, $\Delta E_{\rm phys}(\Gamma)$ is plotted in Supplementary Figure 1.

II. CLASSICAL HOPPING OVER THE BARRIER VERSUS QUANTUM TUNNELING

Two possible mechanisms can explain the experiment described in Fig. 5 of the main text, where the system is initiated at the metastable state and decays to the ground state. The first mechanism is quantum tunneling, where the system tunnels under the energy barrier from the metastable state to the ground state. However, since the system is not perfectly isolated, it is possible that some external noise source makes the system classically hop over the energy barrier from the metastable state to the ground state. To distinguish between the two mechanisms, we

ϵ	Γ/J	Relaxation fraction
0.05	1.4	1
0.0606	1.2	1
0.072	1	0.86
0.0844	0.8	0
0.0973	0.6	0
0.111	0.4	0
0.1255	0.2	0

Supplementary Table I. Fraction of spin configurations managing to decay from the metastable (all-down) to the true ground (all-up) state, performed at different transverse field amplitudes with constant barrier height. All the parameters were kept constant for an annealing schedule of duration 1000 μs. The experiment was repeated 100 times for each set of parameters.

take advantage of the fact that we can independently control two different parameters: the longitudinal magnetic field amplitude ϵ, and the transverse coupling strength Γ. By changing these two factors, we can keep the barrier height constant while changing the transverse field magnitude. If the relaxation mechanism is classical, then to a good approximation the relaxation should only be a function of the barrier height, regardless of the transverse field value. However, for quantum tunneling the magnitude of the transverse field is crucial – for example, one expects zero quantum tunneling for $\Gamma = 0$.

In order to check this, we initiated the system in several initial conditions such that $\Delta E_{\text{phys}}(\Gamma, \epsilon) = \text{const.}$, which based on Eq. (I D) means

$$\epsilon^{-1}\left(1 - \frac{\Gamma}{\Gamma_c}\right)^{2\mu-\beta} = \text{const.} \tag{1}$$

For each pair of (Γ, ϵ) we measured the fraction of systems that were able to relax to the ground state. The results are shown in Table I and show a clear transition occurring around the threshold $\Gamma/J \simeq 1$, above which the decay to the ground state is compatible with tunneling.

III. CLASSICAL MONTE CARLO BASELINE

As a baseline for the experimentally extracted exponents, we performed independent classical Monte Carlo (MC) simulations of the 2D ferromagnetic Ising model on the same lattice geometries used in the quantum-annealer experiments, and applied the same finite-size scaling collapse pipeline and uncertainty estimation. The full simulation and analysis protocol is described in the Methods of the main text; here we report the resulting collapses and bootstrap distributions, together with the comparison to the experimental data.

We simulated the classical 2D Ising ferromagnet with periodic boundary conditions,

$$H(\{\sigma_i\}) = -J\sum_{\langle i,j\rangle}\sigma_i\sigma_j - h\sum_i \sigma_i, \qquad \sigma_i = \pm 1, \tag{2}$$

with $J = 1$ and $h = 0$, on the geometries $(L_x, L_y) \in \{(14, 8), (30, 20), (60, 44)\}$. The exact Onsager critical temperature for $J = 1$ is $T_c = 2/\ln(1 + \sqrt{2})$.

The finite-size scaling collapses obtained from the classical baseline are shown in Supplementary Figure 2, and the bootstrap distributions of the extracted exponents in Supplementary Figure 3. The procedure yields

$$\begin{aligned}\nu &= 0.99 \pm 0.06,\ \text{compatibility} = 0.16\sigma,\\ \beta &= 0.110 \pm 0.007,\ \text{compatibility} = 2.11\sigma,\\ \gamma &= 1.66 \pm 0.09,\ \text{compatibility} = 1.01\sigma.\end{aligned}$$

Two features are worth emphasizing. First, the error bars obtained from the classical baseline are comparable to those of the experiment, and the compatibilities with the exact 2D Ising values are of the same order; in particular, β is the hardest exponent to extract, sitting about 2σ from its exact value in both the classical baseline and the experiment. This is expected: β is small, and the limited per-size statistics amplify the difficulty of its extraction. Second, despite the differences between the two settings—superspin embedding, a small residual transverse field, and generic experimental defects in the annealer—the experiment and the purely classical simulation display strikingly similar behavior in the exponent extraction, including in the structure of the uncertainties. This similarity is a strong indication that the experimental system sits at criticality in the 2D Ising universality class. We note that a

further factor complicating the analysis, common to both the experiment and the classical simulation, is the small variation in lattice aspect ratio L_x/L_y across sizes, dictated by the experimental embedding constraints.

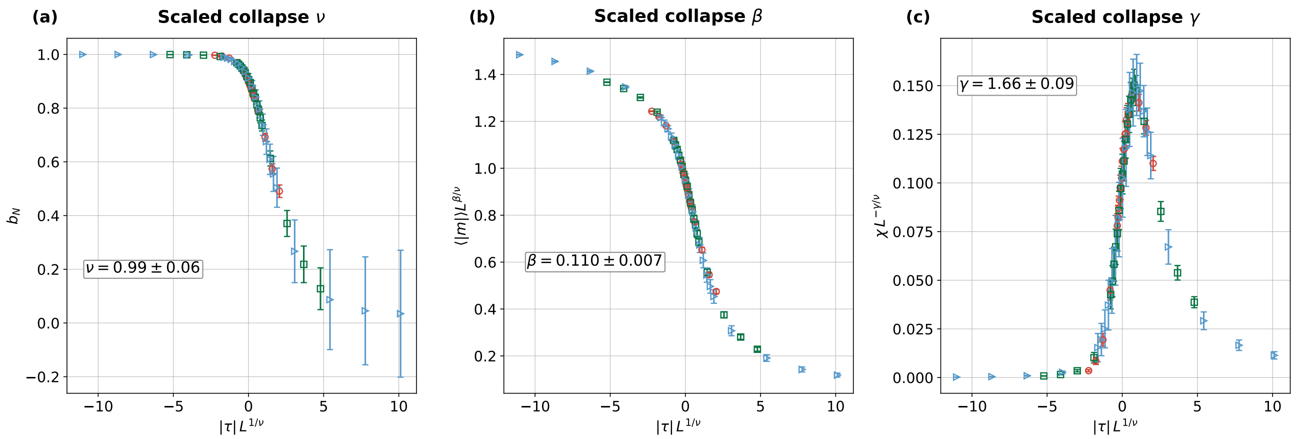

Supplementary Figure 2. **Finite-size scaling collapses for the classical Monte Carlo baseline.** (**a**) Binder cumulant collapse used to extract ν. (**b**) Magnetization collapse used to extract β (using the fitted ν). (**c**) Susceptibility collapse used to extract γ (using the fitted ν). Error bars are standard deviations obtained via the bootstrap procedure described in the main text's Methods.

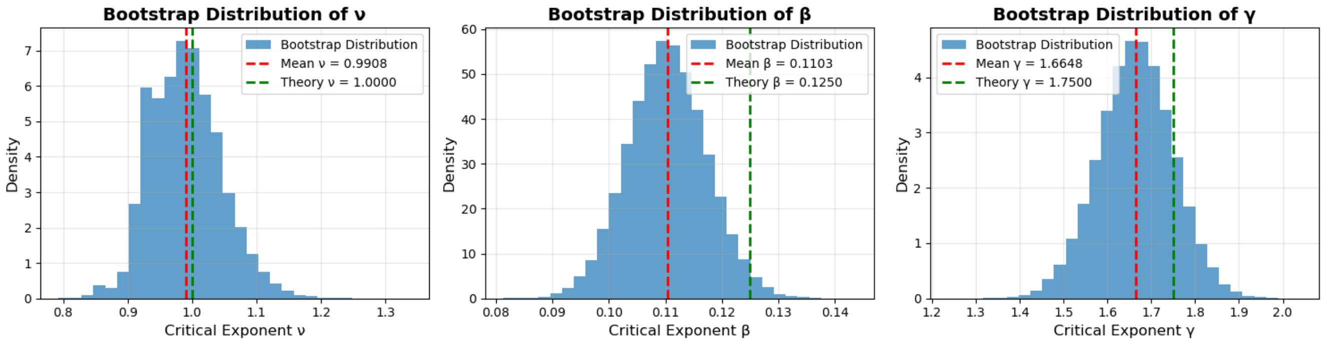

Supplementary Figure 3. **Bootstrap distributions of critical exponents.** Histograms of the bootstrap estimates for ν, β, and γ obtained from the classical Monte Carlo dataset using the same collapse-optimization pipeline as in the experimental analysis. Red dashed lines indicate the bootstrap mean; green dashed lines indicate the exact 2D Ising values.

IV. SHIMMING CONVERGENCE AND EMBEDDING MAPS

The superspin embeddings used to realize the 2D lattice on the quantum annealer, together with the shimming procedure used to calibrate the flux-bias offsets (FBOs) and the gauge randomization used to average out residual biases, are described in detail in the Methods of the main text. Here we report the diagnostics that demonstrate the convergence of the calibration and the robustness of the results across different embeddings.

The convergence of the shimming procedure is illustrated in Supplementary Figure 4, where we show the evolution of the FBOs $\Phi_i^{(n)}$ for all qubits as a function of the iteration number. After a transient regime, all offsets stabilize around well-defined values, indicating convergence of the calibration. The corresponding distribution of final offsets (right panel) is narrow and centered, confirming that residual offsets are small and controlled.

The combination of superspin-level shimming and problem randomization substantially reduces both local and correlated errors induced by the embedding. We have verified that, after these procedures, the observables entering the finite-size scaling analysis are stable across different embeddings and gauge realizations, demonstrating that residual imperfections do not bias the extracted critical behavior. For the smaller and intermediate system sizes, multiple embeddings were implemented across different regions of the QPU (Supplementary Figure 5), allowing us to probe directly the effect of spatial inhomogeneities of the hardware. We find that the Binder cumulants and

the observables entering the finite-size scaling analysis are consistent across these independent embeddings within statistical uncertainties, indicating that embedding-dependent effects do not affect the extracted critical behavior at the level of precision of the present study.

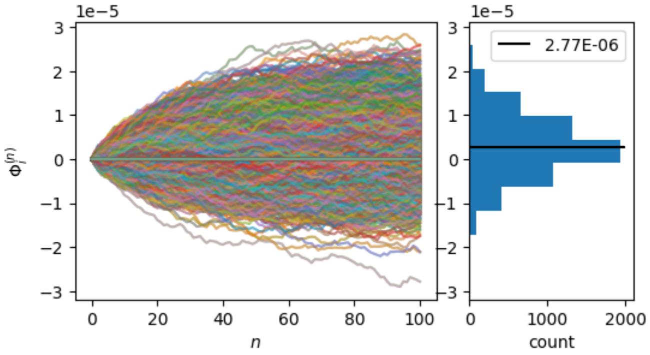

Supplementary Figure 4. Evolution of the flux-bias offsets $\Phi_i^{(n)}$ during the shimming procedure. Iterative trajectories for all qubits, showing convergence to stable values after an initial transient. The distribution of final offsets, demonstrating a narrow spread around a small mean value, is also reported.

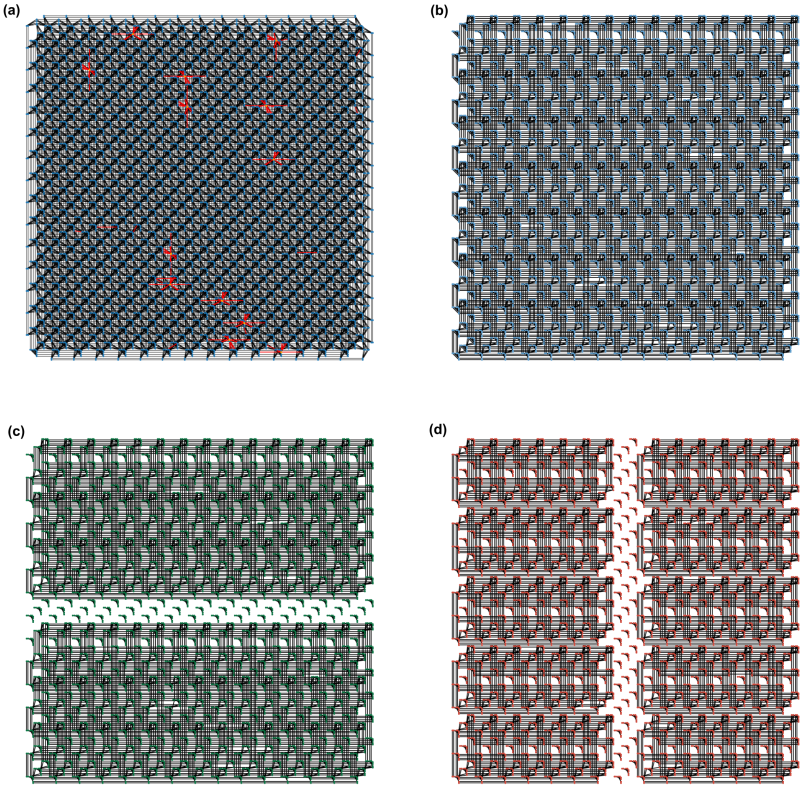

Supplementary Figure 5. Embeddings of the 2D square lattice on a Pegasus graph. Panel **(a)** shows the full `Advantage_system4.1` QPU network. In orange (red) are highlighted defective spins (couplings). Panels **(b)**, **(c)** and **(d)** illustrate the different embeddings used for the 60×44, 30×20 and 14×8 lattices, respectively.

[1]M. Suzuki, Progress of theoretical physics **56**, 1454 (1976).
[2]Z. Friedman, Phys. Rev. B **17**, 1429 (1978).
[3]S. Hesselmann and S. Wessel, Phys. Rev. B **93**, 155157 (2016).
[4]M. Hasenbusch and K. Pinn, Physica A: Statistical Mechanics and its Applications **192**, 342 (1993).
[5]A. Talapov and H. Blöte, Journal of Physics A: Mathematical and General **29**, 5727 (1996).
