# Supplementary material for: Finite-temperature criticality through quantum annealing
Source: Nat Commun. 2026 Jul 23;17:7571. doi: 10.1038/s41467-026-75348-3 (PMC13415517; doi:10.1038/s41467-026-75348-3)
Supplement: Supplementary file 2 — Transparent Peer Review file [file 41467_2026_75348_MOESM2_ESM.pdf]

nature portfolio

Peer Review File

Finite-temperature criticality through quantum annealing

Corresponding Author: Dr Gianluca Teza

This file contains all reviewer reports in order by version, followed by all author rebuttals in order by version.

Version 0:

Reviewer comments:

Reviewer #1

(Remarks to the Author)
This manuscript provides a significant advance in the use of quantum annealers for probing finite-temperature critical phenomena. The embedding strategy, real-time temperature calibration, and scaling analyses are carefully presented, and the agreement with the Onsager solution for the two-dimensional Ising model is impressive. The work will be of broad interest, as it not only addresses a long-standing challenge but also proposes a methodology that could extend to more complex systems.

Nevertheless, there are several points that require more rigorous clarification before publication. First, while the temperature calibration protocol is innovative, its quantitative reliability is not fully established. The manuscript would benefit from a more systematic error analysis, for instance by estimating how much the critical temperature and critical exponents would shift under small miscalibrations of the effective temperature. Without this, it is difficult to assess whether the agreement with theory is truly robust.

Second, the superspin embedding approach, though effective for mitigating edge effects, may itself introduce correlated errors. The discussion of “magnetic leakage” and local field compensation is rather brief; the authors should provide more quantitative evidence that such effects do not bias the scaling analysis, especially for the largest system sizes.

Third, the extracted order parameter exponent $\beta = 0.09 \pm 0.01$ is systematically lower than the exact value of 1/8. While the deviation is small, the authors should explicitly discuss whether this is compatible with finite-size effects in their setup, or whether it reflects residual systematic limitations of the annealer. Some context by comparison with classical Monte Carlo on lattices of the same aspect ratio would be particularly useful.

Finally, while the demonstrations of non-equilibrium relaxation and barrier crossing are appealing, the interpretation in terms of “tunneling” remains qualitative. Given that thermal activation cannot be excluded under the experimental conditions, a clearer discussion of how to distinguish between these mechanisms—or at least the limitations of the present data in making this distinction—would strengthen the claims.

In summary, the manuscript is of high quality and merits publication in Nature Communications, but a more rigorous treatment of calibration accuracy, embedding biases, and universality checks is needed. These clarifications are feasible within the current dataset and would ensure that the results are interpreted with the appropriate level of confidence.

Reviewer #2

(Remarks to the Author)
The authors used a quantum annealer as a Boltzmann sampler to study finite-temperature criticality of the 2S transverse-field Ising model. They achieved this by employing spin embedding, calibrating an effective temperature run by run with a single-qubit thermometer, terminating the anneal early at an intermediate schedule point s* (small A/B), and extracting Tc and critical exponents from Binder cumulants and finite-size scaling.

The main result is that annealer-generated samples reproduce the 2D-Ising universality class at sizeable logical system sizes (up to ~10^3–10^3.5 spins), with a clear Binder-cumulant crossing and exponent estimates β,γ, and ν close to the exact values. This is an interesting demonstration that the quantum annealers can be used to quantitively study phase transitions. I have a few points that, if addressed, would further strengthen the manuscript:

1. Direct evidence of thermalization at s*: As presented, equilibrium at the sampling point is argued indirectly (good Binder crossing and good collapses). For a claim of precision finite-T criticality, I would expect at least one direct equilibrium check at s*: e.g. (i) pause-duration saturation (observables stabilize with longer annealing time), and (ii) single/multi-histogram reweighting of nearby J to a common Teff. Without one such test, a small but systematic deviation from Boltzmann could still appear as clean scaling.

2. Order-parameter exponent β: Despite good collapses and agreements for γ and ν within one standard deviation, the reported $\beta \approx 0.09 \pm 0.01$ is noticeably below the exact 2D-Ising value $1/8 = 0.125$. Please clarify the discrepancy. Cross-checks against hyperscaling relations and alternative estimators would be helpful.

3. Quantitative baseline for dynamics near criticality: The relaxation-with-Gamma and barrier-crossing demonstrations are interesting, but a direct quantitative comparison is still needed to substantiate the claim.

4. Error bars: In the inset of Binder-cumulant crossing (Fig. 2), the error bars were absent. This is essential for judging the robustness of Tc and exponent fits.

Version 1:

Reviewer comments:

Reviewer #1

(Remarks to the Author)
This manuscript addresses the difficult problem of quantitatively probing finite-temperature critical behavior with a quantum annealer. The combination of real-time temperature correction, superspin embedding, toroidal boundary conditions, and finite-size scaling is, in my view, a genuine strength of the work. Demonstrating Binder-cumulant crossings and critical exponents for the 2D Ising ferromagnet, with system sizes extended up to 2640 logical spins, represents a meaningful advance for the field. On balance, I find the study suitable for publication in Nature Communications.

In the revised version, the authors have responded to the previous concerns in a serious and constructive manner. The uncertainty in the temperature calibration is now quantified. To address the deviation in the estimated beta exponent, the authors have added a classical Monte Carlo baseline using the same lattice geometry and the same analysis pipeline. The interpretation of barrier crossing has also been strengthened by the addition of supplementary data obtained at fixed barrier height while varying Gamma/J. These are substantive revisions rather than cosmetic changes, and I appreciate that.

That said, I would still ask the authors to clarify two points before publication. The first concerns systematic uncertainty associated with embedding and gauge choice. The Supplement now provides useful information on the shimming procedure and on the convergence of the flux-bias offsets, but the manuscript would be more convincing if it showed more directly how much the Binder crossings and extracted exponents vary across embeddings and gauges. A brief discussion in the main text would already help, and a small table or figure in the Supplement would be sufficient.

The second point concerns equilibration at s*. The authors use the relaxation measurements in Fig. 4 to argue that the system reaches a steady value within the sampling window, which is helpful. Still, the connection between those measurements and the finite-temperature sampling protocol used for the criticality analysis should be spelled out more clearly. I do not regard further experiments as essential here, but the paper would benefit from a more explicit explanation of what is directly validated by the present protocol and what remains an auxiliary consistency check.

I was initially concerned by the fact that the estimated beta value is lower than the theoretical one. However, the newly added classical Monte Carlo results on the same geometry show a similar tendency, and for that reason I do not think this issue undermines the main conclusions of the paper. Even so, the discussion of universality would be more solid if it relied primarily on the Binder crossing and the consistency of nu and gamma, rather than placing too much weight on the numerical value of beta alone. This only requires a modest adjustment in emphasis.

The discussion around tunneling in Fig. 5 is also interesting. The added comparison at fixed barrier height is useful as supporting evidence that a purely thermal interpretation is not sufficient. At the same time, this part of the paper seems secondary to the main claim, namely the demonstration of finite-temperature criticality. I therefore think the wording should remain somewhat cautious, for example by stating that the observations strongly suggest a quantum contribution, or are consistent with tunneling-assisted relaxation, rather than making a stronger claim than the data can presently sustain.

In summary, I believe this manuscript can be published in Nature Communications. What is still needed is not a major new experimental campaign, but a modest tightening of the presentation and a slightly more careful calibration of the claims.

Reviewer #2

(Remarks to the Author)
The revised manuscript is substantially improved. In particular, the added discussion of the non-equilibrium relaxation experiment on the largest and slowest lattice satisfactorily addresses my main concern regarding equilibration at the sampling point. I now find the equilibrium interpretation sufficiently convincing for the purposes of this work.

The revision also strengthens the manuscript in several other respects. The added uncertainty analysis and the error bars in the Binder-cumulant crossing are helpful, and the supplementary classical Monte Carlo control provides useful context for the low value of β. In my view, this makes it plausible that the remaining deviation in β is at least in part due to geometry and finite-statistics effects rather than indicating a qualitative failure of the protocol. The additional fixed-barrier control in the supplement also improves the metastability discussion.

I therefore support publication after minor revision.

My remaining comments are mainly about wording and emphasis:

1) The claims should be phrased slightly more cautiously in a few places. In particular, expressions such as “definitively demonstrate,” “precisely resolve,” or “full set of critical exponents” read somewhat stronger than necessary, especially given the remaining spread in β.
2) The discussion of the exponents should continue to emphasize that ν and γ are the most robust quantitative benchmarks in the present study, while β remains more sensitive to geometry and statistics.
3) The new supplementary material on shimming and gauge averaging is useful; a brief pointer to this material in the main text would help readers.

Overall, I find that the revision has addressed my main concerns, and I would support publication after minor revision.

Open Access This Peer Review File is licensed under a Creative Commons Attribution 4.0 International License, which permits use, sharing, adaptation, distribution and reproduction in any medium or format, as long as you give appropriate credit to the original author(s) and the source, provide a link to the Creative Commons license, and indicate if changes were made.
In cases where reviewers are anonymous, credit should be given to 'Anonymous Referee' and the source.
The images or other third party material in this Peer Review File are included in the article’s Creative Commons license, unless indicated otherwise in a credit line to the material. If material is not included in the article’s Creative Commons license and your intended use is not permitted by statutory regulation or exceeds the permitted use, you will need to obtain permission directly from the copyright holder.
To view a copy of this license, visit https://creativecommons.org/licenses/by/4.0/

Point-by-point response to Reviewer #1

We thank Referee 1 for the time and the detailed remarks. Below we provide a detailed point-by-point reply, while at the end follows a copy of the revised manuscript witht he new relevant additions and edits highlighted in blue.

"This manuscript provides a significant advance in the use of quantum annealers for probing finite-temperature critical phenomena. The embedding strategy, real-time temperature calibration, and scaling analyses are carefully presented, and the agreement with the Onsager solution for the two-dimensional Ising model is impressive. The work will be of broad interest, as it not only addresses a long-standing challenge but also proposes a methodology that could extend to more complex systems."

We thank the referee for this positive assessment of our work. We are pleased that the referee recognizes it as a significant advance in the use of quantum annealers to probe finite-temperature critical phenomena and finds the agreement with the Onsager solution compelling. We also appreciate the observation that the methodology may be applicable to more complex systems and therefore of broad interest. These remarks encourage us that the scope and impact of the results are clearly conveyed.

"Nevertheless, there are several points that require more rigorous clarification before publication. First, while the temperature calibration protocol is innovative, its quantitative reliability is not fully established. The manuscript would benefit from a more systematic error analysis, for instance by estimating how much the critical temperature and critical exponents would shift under small miscalibrations of the effective temperature. Without this, it is difficult to assess whether the agreement with theory is truly robust."

We thank the referee for requesting a quantitative assessment of the temperature calibration accuracy. We had performed an estimate of the uncertainty on a single temperature measurement, but we did not report an explicit uncertainty in the original manuscript because the calibration error is much smaller than the statistical uncertainty arising from finite sampling.

Indeed, the effective temperature is estimated through single-qubit freeze-out measurements performed across the entire QPU (more than 5×10^3 qubits) with 10^3 repetitions, yielding over 5×10^6 data points for each calibration run (see the following D-Wave ducumentation link). The resulting relative uncertainty of a single temperature estimate is of order $\sim 10^{-3}$. Propagating such a variation through the finite-size scaling analysis produces shifts in T_c and in the critical exponents well below the statistical error bars.

Additionally, the stability of the Binder-cumulant crossings across consecutive experiments further confirms that relevant QPU temperature drifts occur on timescales long compared to the duration of a sampling experiment performed immediately after calibration.

We emphasize that the primary role of the calibration protocol is to compensate for large hardware temperature fluctuations and freeze-out variations. Without this procedure Binder crossings are not observable, preventing any reliable scaling analysis at large sizes. The protocol could be further refined, for instance by characterizing possible spatial inhomogeneities, which we plan to investigate in future dedicated work. The manuscript now clarifies these considerations on the single measurement of the QPU temperature more in detail (see the new addition highlighted in blue in the **"Finite-temperature sampling" section of the main text**).

"Second, the superspin embedding approach, though effective for mitigating edge effects, may itself introduce correlated errors. The discussion of "magnetic leakage" and local field compensation is rather brief; the authors should provide more quantitative evidence that such effects do not bias the scaling analysis, especially for the largest system sizes."

We agree with the referee that embedding-induced correlated errors must be carefully controlled. In the revised Supplementary Information (**newly added Section V**) we now provide a detailed description of the shimming procedure and of the problem-isomorphism strategy used to compensate local field offsets and suppress magnetic leakage effects.

In particular, we clarify how gauge averaging and embedding symmetrization are employed to remove systematic biases and we show that observables entering the scaling analysis remain stable across different embeddings and gauges. This demonstrates that residual leakage effects do not affect the extracted critical behavior, including for the largest system sizes considered.

"Third, the extracted order parameter exponent $\beta = 0.09 \pm 0.01$ is systematically lower than the exact value of 1/8. While the deviation is small, the authors should explicitly discuss whether this is compatible with finite-size effects in their setup, or whether it reflects residual systematic limitations of the annealer. Some context by comparison with classical Monte Carlo on lattices of the same aspect ratio would be particularly useful."

We thank the referee for highlighting this point. The order-parameter exponent β is particularly challenging to extract experimentally because its exact value in the 2D Ising universality class ($\beta = 1/8$) is small, making it highly sensitive to finite-size corrections and statistical fluctuations. The measured value $\beta = 0.09 \pm 0.01$ remains around two standard deviations of the exact prediction.

Several systematic effects contribute to a residual shift. The main factor, as we discussed in what follows, has to do with the aspect ratio that cannot be kept strictly constant across all programmable sizes. This mixes slightly different finite-size scaling functions and can effectively renormalize the fitted β. Additional factor is the available statistics, which introduce additional bias in a quantity determined from a narrow critical region. Lastly, the implemented Hamiltonian contains a small but finite transverse field, so the system realizes a transverse-field Ising model rather than a purely classical one; this produces size-dependent corrections to scaling.

To explore the main reasons for the bias in β, in the revised Supplementary Information we now include a dedicated classical Monte Carlo baseline section in which we simulate the 2D Ising ferromagnet on the same rectangular lattices and with the same boundary conditions used in the experiments, namely $(L_x, L_y) \in \{(14,8),(30,20),(60,44)\}$ with periodic boundary conditions.

We then process the classical data with exactly the same finite-size collapse optimization pipeline used for the quantum-annealer data, and we emulate the different experimental sample counts by bootstrap downsampling the classical dataset to the same effective statistics ($N_{\mathrm{exp}} = 1000$, 400, 100 reads for the three sizes). This yields classical exponent estimates

$$\nu = 0.99 \pm 0.06, \qquad \beta = 0.110 \pm 0.007, \qquad \gamma = 1.66 \pm 0.09,$$

This analysis demonstrates that the estimated value of β is more sensitive to the changes in the lattice geometry, as well as the limited statistics. Moreover, in both the MC simulation and the D-wave experiment, the estimated value of β is about 2σ *lower* than the theoretical value. These findings are discussed in a dedicated section in the Supplemental Information (see the **newly added Section IV**) and briefly discussed in the main text as well.

"Finally, while the demonstrations of non-equilibrium relaxation and barrier crossing are appealing, the interpretation in terms of "tunneling" remains qualitative. Given that thermal activation cannot be excluded under the experimental conditions, a clearer discussion of how to distinguish between these mechanisms—or at least the limitations of the present data in making this distinction—would strengthen the claims."

We thank the referee for raising this important point, also noted by Reviewer 2. The key question is whether the observed relaxation from the metastable configuration can be attributed solely to thermally activated barrier crossing or requires a tunneling-assisted mechanism.

In the original manuscript we estimated the barrier height to be approximately $60\,k_B T$, implying a purely thermal hopping rate $r \sim e^{-E/k_B T} \sim 10^{-26}$, and therefore negligible on experimental timescales. However, one can claim that an unknown pre-factor might compensate for this tiny hopping rate. To further clarify the mechanism, we performed an additional series of experiments in which the energy bias ϵ between the wells is varied while keeping the barrier height fixed and tuning the transverse-field ratio Γ/J.

In the revised Supplementary Information we report (in the **newly added Section II**) the fraction of configurations prepared in the metastable well that relax to the ground state after a $1000\,\mu$s anneal. We observe a clear threshold around $\Gamma/J \simeq 1$: below this value relaxation is strongly suppressed, while above it the system efficiently reaches the ground state. Since the temperature and barrier height are unchanged, such behavior is incompatible with purely thermally activated dynamics, but is consistent with tunneling-assisted relaxation.

We have revised the manuscript to reflect this more precise interpretation and to explicitly state the limits of what can be inferred from the present data. A more detailed quantitative study of the crossover between activation and tunneling is an interesting direction for future work.

"In summary, the manuscript is of high quality and merits publication in Nature Communications, but a more rigorous treatment of calibration accuracy, embedding biases, and universality checks is needed. These clarifications are feasible within the current dataset and would ensure that the results are interpreted with the appropriate level of confidence."

We thank the referee for the careful reading and constructive remarks. The requested clarifications regarding calibration accuracy, embedding effects, and universality analysis have been incorporated into the revised manuscript and Supplementary Information. We believe these additions improve both the clarity of the presentation and the robustness of the conclusions.

Point-by-point response to Reviewer #2

We thank Referee 1 for the time and the detailed remarks. Below we provide a detailed point-by-point reply, while at the end follows a copy of the revised manuscript witht he new relevant additions and edits highlighted in blue.

"The authors used a quantum annealer as a Boltzmann sampler to study finite-temperature criticality of the 2S transverse-field Ising model. They achieved this by employing spin embedding, calibrating an effective temperature run by run with a single-qubit thermometer, terminating the anneal early at an intermediate schedule point s (small A/B), and extracting Tc and critical exponents from Binder cumulants and finite-size scaling. The main result is that annealer-generated samples reproduce the 2D-Ising universality class at sizeable logical system sizes (up to* $\sim 10^3$*˘*$10^{3.5}$ *spins), with a clear Binder-cumulant crossing and exponent estimates* β*,*γ*, and* ν *close to the exact values. This is an interesting demonstration that the quantum annealers can be used to quantitively study phase transitions. I have a few points that, if addressed, would further strengthen the manuscript:"*

We thank the referee for the careful summary of our work and for recognizing it as an interesting demonstration of using quantum annealers to quantitatively study finite-temperature phase transitions. We also appreciate the constructive suggestions, which helped us further strengthen the manuscript. We address the specific points raised below.

"1. Direct evidence of thermalization at s: As presented, equilibrium at the sampling point is argued indirectly (good Binder crossing and good collapses). For a claim of precision finite-T criticality, I would expect at least one direct equilibrium check at s*: e.g. (i) pause-duration saturation (observables stabilize with longer annealing time), and (ii) single/multi-histogram reweighting of nearby J to a common Teff. Without one such test, a small but systematic deviation from Boltzmann could still appear as clean scaling."*

We thank the referee for suggesting a direct equilibrium check, but this point can be directly addressed by the set of non-equilibrium experiments performed for analyzing the thermalization of the order parameter (i.e. magnetization) at different transverse field amplitudes (Fig. 4 of the manuscript).

The non-equilibrium relaxation experiments are performed on the largest lattice considered, corresponding to the slowest intrinsic dynamics. Following a quench from the disordered phase, we monitor the total magnetization and observe that it reaches a stationary value within the $1000\,\mu$s annealing time used for the sampling experiments. In particular, at $\Gamma/J \simeq 0.5$—deep in the ordered phase—the order parameter stabilizes rapidly even under this extreme quench protocol.

Since equilibration occurs on the largest and slowest system within the experimental time window, this provides direct evidence that the configurations sampled at the measurement point effectively correspond to thermal equilibrium.

"2. Order-parameter exponent β*: Despite good collapses and agreements for* γ *and* ν *within one standard deviation, the reported* $\beta \approx 0.09 \pm 0.01$ *is noticeably below the exact 2D-Ising value* $1/8 = 0.125$*. Please clarify the discrepancy. Cross-checks against hyperscaling relations and alternative estimators would be helpful."*

We thank the referee for highlighting this discrepancy. We address the origin of the small shift in β in detail in our response to Reviewer #1 (third comment), where we discuss slight aspect-ratio variations across programmable sizes, limited statistics, and compared our results to MC simulations on similar system. We have also clarified this discussion in the revised manuscript and Supplementary Information (see the **newly added Section IV**), to explain why β is the most sensitive exponent in our analysis, while ν and γ remain consistent with the 2D Ising universality class.

"3. Quantitative baseline for dynamics near criticality: The relaxation-with-Gamma and barrier-crossing demonstrations are interesting, but a direct quantitative comparison is still needed to substantiate the claim."

We thank the referee for this suggestion. We address this point in detail in our response to Reviewer #1 (last comment), where we introduce an additional experiment in which the barrier height is kept fixed while varying Γ/J. The observed threshold behavior provides a quantitative criterion distinguishing thermally activated dynamics from tunneling-assisted relaxation within the accessible regime. We have clarified this analysis in the revised manuscript and Supplemental Information (in the **newly added Section II**).

"4. Error bars: In the inset of Binder-cumulant crossing (Fig. 2), the error bars were absent. This is essential for judging the robustness of Tc and exponent fits."

We thank the referee for pointing out this omission. Error bars have now been included in the Binder-cumulant inset in the revised manuscript, allowing a direct assessment of the robustness of the extracted T_c and critical exponents.

Point-by-point response to the second report of Reviewer #1

We thank Referee 1 and for finding that we *"replied in a serious and constructive manner"* and appreciating our revisions to be *"substantive rather than cosmetic changes"*. We also thank the Referee for finding *"the study suitable for publication in Nature Communications"* and for taking the time to provide additional suggestions to improve the exposition, which we address below. At the end follows a copy of the revised manuscript and SM with the new relevant additions and edits highlighted in blue.

" ...That said, I would still ask the authors to clarify two points before publication. The first concerns systematic uncertainty associated with embedding and gauge choice. The Supplement now provides useful information on the shimming procedure and on the convergence of the flux-bias offsets, but the manuscript would be more convincing if it showed more directly how much the Binder crossings and extracted exponents vary across embeddings and gauges. A brief discussion in the main text would already help, and a small table or figure in the Supplement would be sufficient. "

We thank the reviewer for this important point. We have clarified both aspects raised.

(i) Embedding robustness. For the smaller and intermediate system sizes, we implemented multiple embeddings distributed across different regions of the QPU. These independent embeddings were used both to increase statistics and to verify robustness against spatial inhomogeneities of the hardware. We find that the Binder cumulants and observables entering the finite-size scaling analysis are consistent across these embeddings within the statistical uncertainty.

(ii) Gauge transformations. Gauge transformations (spin+couplings reversal operations) were randomized independently at each experimental run. Since the ferromagnetic Ising model is invariant under such transformations (upon appropriate decoding), this procedure preserves physical observables while averaging over residual hardware biases. Randomizing the gauge at each run ensures effective symmetrization over device imperfections.

To make this explicit, we have:

- Added a short clarification in the main text referring to the Supplementary Material where needed;
- Included a new figure in Section V of the Supplementary Material showing the different embeddings across the QPU and demonstrating coverage of distinct regions.

"The second point concerns equilibration at s. The authors use the relaxation measurements in Fig. 4 to argue that the system reaches a steady value within the sampling window, which is helpful. Still, the connection between those measurements and the finite-temperature sampling protocol used for the criticality analysis should be spelled out more clearly. I do not regard further experiments as essential here, but the paper would benefit from a more explicit explanation of what is directly validated by the present protocol and what remains an auxiliary consistency check."*

We agree and have clarified this point. In the revised version we now explicitly state that the relaxation measurements provide an auxiliary consistency check, demonstrating that the system reaches a steady response within the experimental timescale in the relevant operating regime. At the same time, we clarify that these measurements do not by themselves prove equilibrium sampling for the criticality analysis.

We emphasize that the primary validation of the equilibrium interpretation is provided by: (i) the Binder-cumulant crossing across system sizes, (ii) the agreement of the crossing value with known results, (iii) the consistency of the finite-size scaling collapse.

"I was initially concerned by the fact that the estimated beta value is lower than the theoretical one. However, the newly added classical Monte Carlo results on the same geometry show a similar tendency, and for that reason I do not think this issue undermines the main conclusions of the paper. Even so, the discussion of universality would be more solid if it relied primarily on the Binder crossing and the consistency of nu and gamma, rather than placing too much weight on the numerical value of beta alone. This only requires a modest adjustment in emphasis. "

We agree and have revised the discussion accordingly.

In the revised manuscript, we now:

- emphasize the Binder crossing and the agreement of ν and γ as the most robust indicators of universality;
- present β more cautiously, explaining the difficulty in estimating such a small (1/8) exponent given the limiting experimental constraints (samples statistics and embedding geometries), consistent with both experimental data and the classical Monte Carlo baseline.

"The discussion around tunneling in Fig. 5 is also interesting. The added comparison at fixed barrier height is useful as supporting evidence that a purely thermal interpretation is not sufficient. At the same time, this part of the paper seems secondary to the main claim, namely the demonstration of finite-temperature criticality. I therefore think the wording should remain somewhat cautious, for example by stating that the observations strongly suggest a quantum

contribution, or are consistent with tunneling-assisted relaxation, rather than making a stronger claim than the data can presently sustain. "

We agree and have softened the language accordingly. In the revised version it is stated that the observations are consistent with tunneling-assisted relaxation and provide strong evidence against a purely thermal mechanism, rather than claiming a definitive demonstration of quantum tunneling.

Point-by-point response to the second report of Reviewer #2

We thank Referee 1 for finding the *"revised manuscript is substantially improved"* and that the additions address all their concerns and improve the discussion. Lastly, we thank the Referee for supporting *"publication after minor revision"*, which we highlight in detail below with a point-by-point reply to their last comments and a copy of the revised manuscript and SM with the new relevant additions and edits highlighted in blue.

"1) The claims should be phrased slightly more cautiously in a few places. In particular, expressions such as "definitively demonstrate," "precisely resolve," or "full set of critical exponents" read somewhat stronger than necessary, especially given the remaining spread in β"

We agree and have moderated the wording throughout the manuscript.

"2) The discussion of the exponents should continue to emphasize that ν and γ are the most robust quantitative benchmarks in the present study, while β remains more sensitive to geometry and statistics."

We have revised the discussion accordingly, explicitly identifying ν and γ as the most robust benchmarks, while presenting β as more sensitive to geometry and statistics.

"3) The new supplementary material on shimming and gauge averaging is useful; a brief pointer to this material in the main text would help readers."

We have added an explicit pointer in the main text to the Supplementary Material, where shimming, gauge transformations, and embedding robustness are described in detail.
